# Dietary Sodium and Human Health

**DOI:** 10.3390/nu15173696

**Published:** 2023-08-24

**Authors:** David A. Jaques, Belen Ponte

**Affiliations:** Division of Nephrology and Hypertension, Department of Medicine, Geneva University Hospitals, Rue Gabrielle-Perret Gentil 4, 1205 Geneva, Switzerland

Sodium, contained in dietary salt, is essential to human life. For millions of years, our ancestors did not add salt to their diet but rather relied on sodium naturally present in unprocessed food, corresponding to a daily load of less than 0.5 g of salt. As a minimal amount of sodium is needed to maintain homeostasis, powerful mechanisms to retain sodium were developed throughout evolution. A few thousand years ago, however, salt became a highly valued trade item because it allowed food preservation and transportation over large distances. Salt consumption drastically increased worldwide, to approach 10 g per day in most countries. This pronounced increase in salt consumption over a relatively short time span poses a significant challenge in evolutionary terms. Consequently, dietary sodium excess is thought to negatively impact human health, with millions of deaths indirectly attributed to high salt consumption each year. This Special Issue features research conducted by various groups focusing on the broad topic of “Dietary Sodium and Human Health”. It comprises six original articles and one narrative review that, together, tackle the intricate interplay between salt consumption and human health from the molecular up to the populational level.

While high salt consumption is recognized by most as a causal factor in cardiovascular morbidity and mortality, the benefit of very low sodium intake (<5 g/day) has been debated, with observational data reporting higher all-cause and cardiovascular mortality in those patients. To untangle this apparent paradox, Burnier et al. analyzed a population-based survey conducted between 2010 and 2012 in the Swiss adult population [1]. They found that only 13.3% of the Swiss population consumed less than 5 g of salt per day. While not being more aware of the effects of salt on health, people with limited salt consumption had lower blood pressure (BP) and body mass index as well as lower cigarette and alcohol consumption. Overall, authors could not identify factors linked to a higher cardiovascular risk in low salt consumers. This populational study thus offers indirect arguments against a potential J-shape association between sodium intake and cardiovascular events and supports the view of residual confounding and/or reverse causality linking low sodium intake to adverse events.

Sodium is not the only nutrient potentially modulating cardiovascular risk, and observational evidence suggests that sodium and potassium intakes should be interpreted jointly. In parallel with a decrease in salt consumption, an increase in potassium intake is thus recommended by the World Health Organization (WHO). Furthermore, epidemiological studies have highlighted the existence of socioeconomic and geographic disparities in sodium as well as potassium intakes. To better characterize those patterns, De Ridder et al. used spatial-based information from a population survey including more than 20,000 participants in the canton of Geneva, Switzerland [2]. The authors found a clear spatial clustering of the sodium/potassium intake ratio, with socio-demographic characteristics explaining a large part of this effect. These findings suggest the need for public health interventions tailored towards specific populations rather than the large-scale implementation of generic guidelines.

Beyond the effects on BP and cardiovascular prognosis, sodium homeostasis is also central to the development of hyponatremia, the most common electrolyte imbalance. Independently of its causes, hyponatremia has been associated with various adverse outcomes such as falls, fractures, prolonged hospitalization, and mortality risk in the general population. Whether hyponatremia is a causal factor in this setting or simply an indirect marker of underlying comorbidities is debated. In liver transplant candidates, hyponatremia is associated with mortality as well as poor graft survival after transplantation [3]. Using a Swiss prospective cohort of 1315 kidney recipients, Berchtold et al. found that hyponatremia at 6 months after transplantation affected 7.4% of the population but was not associated with mortality or a composite outcome of rapid decline in kidney function, graft loss or mortality. These results contrast with a previous study from Korea that reported an increased risk of graft failure and mortality in patients with hyponatremia at 3 months after kidney transplant [4]. These differences are likely explained by distinct populations and methodologies, while residual confounding might also play a role.

On a pathophysiological level, multiple intricate mechanisms are responsible for the association between sodium intake, hypertension, and cardiovascular events. While the effects of sodium content on blood volume, and thus BP, are relatively straightforward, the activity of renin-angiotensin (RAS) and sympathetic nervous (SNS) systems are also important contributors to the development of hypertensive disorders. Moreover, experimental evidence also points towards the direct adverse consequences of sodium load on endothelial function, independent of BP. Given this intricate interplay between several systems, Stupin et al. investigated the effect of salt loading on vascular function while measuring SNS activity in 47 young healthy individuals sequentially submitted to 7-day low- and high-salt diets [5]. SNS activity was assessed through 24 h urine catecholamine excretion and endothelial function with skin post-occlusive reactive hyperemia and acetylcholine-induced dilatation. Authors found that short-term salt loading suppressed SNS activity and impaired vascular reactivity. Furthermore, the exogenous modulation of SNS activity by a mental stress test did not directly impact endothelial function. They concluded that the suppression of SNS during salt loading represents a physiological adaptation, rather than a pathophysiological mechanism by which sodium would affect vascular function. They postulate that the switch from (physiological) SNS suppression to (pathological) SNS activation in response to salt loading could represent the basis of the salt-sensitivity phenomenon in response to dietary salt intake.

On the other end of the spectrum, it has been suggested that dietary sodium restriction could have a negative impact on lipid and glucose metabolism in animal as well as in human studies [6,7]. It is thought that long-term sodium restriction could activate RAS and SNS systems, thereby favoring insulin resistance, hyperlipidemia, and lipid infiltration in arterial walls. In an experimental study, da Silva Ferreira et al. investigated the effect of aerobic exercise training on LDL-knock-out mice fed with a low- or normal salt diet [8]. Intensive dietary sodium restriction negatively impacted markers of glucose and lipid metabolism in the peripheral blood. In the liver, the low salt diet also favored triglyceride accumulation in association with a corresponding shift in gene expression. Most importantly, however, peripheral insulin resistance and fat accumulation in the liver were entirely negated by aerobic exercise training in those mice.

Beyond its impact on hemodynamic, vascular, and metabolic properties, salt intake could also mediate target organ damage through the modulation of immune pathways. To further test this hypothesis, Bier et al. submitted salt-sensitive rats to a high-salt diet with or without melatonin [9]. While salt loading increased mortality and kidney T-cell infiltration in vivo, as well as T-cell chemoattractant expression in vitro, treatment with melatonine could mitigate those effects. Those results support recent experimental evidence showing that T-cell-deficient rats were protected from salt-induced hypertension [10]. More generally, it points toward immunological properties of salt on the kidney that could be modulated by melatonin treatment in the setting of hypertension.

Finally, this “Dietary Sodium and Human Health” Special Issue is closed by a narrative review on sodium intake as a potential cardiovascular risk factor [11]. Based on available evidence, the authors argue in favor of a causal role of salt consumption in cardiovascular prognosis. It is postulated that those effects are mediated not only by BP modulation, but also by pathways involving direct detrimental consequences of high sodium intake. Overall, while individual responses may vary, sodium intake should be perceived as a cardiovascular risk factor and the reduction of its consumption on a population level represents a potential strategy to decrease the burden of cardiovascular disease worldwide.

## References

[1] Burnier M., Paccaud F.M., Bochud M. (2020). Clinical profiles and factors associated with a low sodium intake in the population: An analysis of the swiss survey on salt. Nutrients.

[2] De Ridder D., Belle F.N., Marques-vidal P., Ponte B., Bochud M., Stringhini S., Joost S., Guessous I. (2021). Geospatial analysis of sodium and potassium intake: A swiss population-based study. Nutrients.

[3] Hackworth W.A., Heuman D.M., Sanyal A.J., Fisher R.A., Sterling R.K., Luketic V.A., Shiffman M.L., Maluf D.G., Cotterell A.H., Posner M.P. (2009). Effect of hyponatraemia on outcomes following orthotopic liver transplantation. Liver Int..

[4] Han S.S., Han M., Park J.Y., An J.N., Park S., Park S.K., Han D.J., Na K.Y., Oh Y.K., Lim C.S. (2016). Posttransplant hyponatremia predicts graft failure and mortality in kidney transplantation recipients: A multicenter cohort study in Korea. PLoS ONE.

[5] Stupin A., Drenjančević I., Šušnjara P., Debeljak Ž., Kolobarić N., Jukić I., Mihaljević Z., Martinović G., Selthofer-Relatić K. (2021). Is there association between altered adrenergic system activity and microvascular endothelial dysfunction induced by a 7-day high salt intake in young healthy individuals. Nutrients.

[6] Catanozi S., Rocha J.C., Nakandakare E.R., Passarelli M., Mesquita C.H., Silva A.A., Dolnikoff M.S., Harada L.M., Quintão E.C.R., Heimann J.C. (2001). The rise of the plasma lipid concentration elicited by dietary sodium chloride restriction in Wistar rats is due to an impairment of the plasma triacylglycerol removal rate. Atherosclerosis.

[7] Nakandakare E.R., Charf A.M., Santos F.C., Nunes V.S., Ortega K., Lottenberg A.M.P., Mion D., Nakano T., Nakajima K., D’Amico E.A. (2008). Dietary salt restriction increases plasma lipoprotein and inflammatory marker concentrations in hypertensive patients. Atherosclerosis.

[8] Da Silva Ferreira G., Bochi A.P.G., Pinto P.R., Del Bianco V., Rodrigues L.G., Morais M.R.P.T., Nakandakare E.R., Machado U.F., Catanozi S., Passarelli M. (2021). Aerobic exercise training prevents insulin resistance and hepatic lipid accumulation in ldl receptor knockout mice chronically fed a low-sodium diet. Nutrients.

[9] Bier A., Khashab R., Sharabi Y., Grossman E., Leibowitz A. (2021). Melatonin prevents T lymphocyte infiltration to the kidneys of hypertensive rats, induced by a high-salt diet, by preventing the expression of CXCR3 ligand chemokines. Nutrients.

[10] Fehrenbach D.J., Dasinger J.H., Lund H., Zemaj J., Mattson D.L. (2020). Splenocyte transfer exacerbates salt-sensitive hypertension in rats. Exp. Physiol..

[11] Jaques D.A., Wuerzner G., Ponte B. (2021). Sodium intake as a cardiovascular risk factor: A narrative review. Nutrients.

